# The Developmental Regulator Pax6 Is Essential for Maintenance of Islet Cell Function in the Adult Mouse Pancreas

**DOI:** 10.1371/journal.pone.0054173

**Published:** 2013-01-11

**Authors:** Alan W. Hart, Sebastien Mella, Jacek Mendrychowski, Veronica van Heyningen, Dirk A. Kleinjan

**Affiliations:** Medical Research Council Human Genetics Unit at the Institute of Genetics and Molecular Medicine at the University of Edinburgh, Edinburgh, United Kingdom; Rutgers University, United States of America

## Abstract

The transcription factor Pax6 is a developmental regulator with a crucial role in development of the eye, brain, and olfactory system. Pax6 is also required for correct development of the endocrine pancreas and specification of hormone producing endocrine cell types. Glucagon-producing cells are almost completely lost in Pax6-null embryos, and insulin-expressing beta and somatostatin-expressing delta cells are reduced. While the developmental role of Pax6 is well-established, investigation of a further role for Pax6 in the maintenance of adult pancreatic function is normally precluded due to neonatal lethality of Pax6-null mice. Here a tamoxifen-inducible ubiquitous Cre transgene was used to inactivate Pax6 at 6 months of age in a conditional mouse model to assess the effect of losing Pax6 function in adulthood. The effect on glucose homeostasis and the expression of key islet cell markers was measured. Homozygous Pax6 deletion mice, but not controls, presented with all the symptoms of classical diabetes leading to severe weight loss requiring termination of the experiment five weeks after first tamoxifen administration. Immunohistochemical analysis of the pancreata revealed almost complete loss of Pax6 and much reduced expression of insulin, glucagon, and somatostatin. Several other markers of islet cell function were also affected. Notably, strong upregulation in the number of ghrelin-expressing endocrine cells was observed. These findings demonstrate that Pax6 is essential for adult maintenance of glucose homeostasis and function of the endocrine pancreas.

## Introduction

Maintenance of glucose homeostasis is essential for life and health. The regulation of blood sugar levels is achieved by peptide hormone secretion from the endocrine cells of the pancreas, located in the islets of Langerhans. Dysfunction or destruction of pancreatic β-cells leads to diabetes mellitus, and better understanding of the factors required for maintenance of functional islet cells is of great medical importance. Over the past two decades the identity and role of different transcription factors and cell markers for pancreas development has become increasingly well-defined, and significant insight has been achieved into the combinations and interactions of regulatory factors required at different stages [1]–[3]. The paired and homeodomain containing transcription factor *Pax6* plays a key role in the development of the endocrine pancreas, in addition to its more widely known role in eye, brain and olfactory system development [4]. Well-defined disease phenotypes have been described in mammals carrying heterozygous loss of function mutations. Homozygotes die neonatally with absent eyes and olfactory bulbs, with brain malformations, as well as pancreatic insufficiency [5], [6]. *Pax6* is required for the correct development and spatial organisation of all islet cell precursors [6], [7], following full endocrine progenitor cell commitment triggered by the expression of high levels of *Ngn3* [8]. *Pax6* has been shown to be regulated by *Pdx1* [9] and in zebrafish the *pax6b* gene was demonstrated to be a direct pdx1 target [10]. *Pax6* is subsequently required for the differentiation of alpha cells [11]. Once the different endocrine cell types of the islet are defined, Pax6 expression is necessary to control the precise expression of each endocrine hormone: glucagon, insulin, somatostatin and pancreatic polypeptide [7], [12]. The more recently identified epsilon cells, derived from Ngn3-positive endocrine precursors that have turned off Pax4 and Nkx2.2 [13], [14], produce the 28 amino acid orexigenic peptide ghrelin. A proportion of alpha cells co-express ghrelin with glucagon. All the endocrine cells carry secretagogue receptors for ghrelin, which modulate endocrine hormone production. Towards the end of the fetal stage the pancreas is the major source of ghrelin. Ghrelin is also produced in the stomach, intestine and brain [15], and in adult life these tissues are the major sites of production, while there remain very few, if any ghrelin producing cells in the adult pancreas [14]. Ghrelin-expressing cells are greatly increased while different endocrine cell types are depleted in the developing pancreas of Pax6-null, Pax4-null or Nkx2.2-null mice [13], [14].

It is becoming increasingly clear that many key developmental regulators fulfill multiple roles at different stages of development, in the progression from initiation to commitment, differentiation and maturation of cell types [3], [16]. Once development has been completed and the adult complement of cells established, a requirement for tissue maintenance and regeneration may persist. The later role of developmentally acting transcription factors that continue to be expressed into adulthood remains poorly explored. However, there is evidence, for example for Pdx1 [17] and for the non-pancreas-expressed gene Sox2 [18], that such genes also function in tissue maintenance. Pax6 is expressed in a number of adult tissues, including pancreatic islet cells [19], [20], [21], as well as brain [22], olfactory [23] and ocular cells [24]. Functional deficits in PAX6-expressing tissues have been observed in patients with PAX6 haploinsufficiency, most notably associated with the developmental eye anomaly aniridia (absence of the iris), where corneal limbal stem cell defects [25] and deficits of olfactory function and neurological organisation [26] have been reported [22]. There have also been reports of glucose intolerance and predisposition to diabetes in some individuals, and in some rodent models, with heterozygous *PAX6* mutations [19], [20], [21], [27], [28]. Because of the multiple essential functions fulfilled by Pax6 during development, it is difficult to dissect its role in adult tissues where Pax6 expression is maintained.

Here we have made use of a conditional *Pax6* mouse mutant in which exons 5–6 are flanked by LoxP sites [29], crossed with an inducible Cre deletor strain [30] to study the potential role of Pax6 in adult tissue maintenance. Cre-mediated deletion of Pax6 in healthy adult mice was activated at 6 months of age. We show that the loss of Pax6 leads to a rapid appearance of diabetic symptoms and strong deterioration in health within a few weeks. A number of pancreatic hormones, transcription factors and insulin processing enzymes are affected by the loss of Pax6, demonstrating an essential role for Pax6 in the continued maintenance of pancreas function in adult life.

## Materials and Methods

### Ethics Statement

This study was approved by the University of Edinburgh ethical committee (approval ID TR-18-07) and performed under UK Home Office license number PPL 60/3785. Animals were cared for in accordance with the guidelines for animal care and experimentation of the University of Edinburgh. All animals were monitored twice daily by visual inspection and by weekly weight measurements. Every effort was made to minimize suffering. The experiment was terminated when weight loss in the CPTD6 (Cre™-Pax6 floxed-Tamoxifen-Diabetic) mice approached 20% of starting body weight.

**Figure 1:**
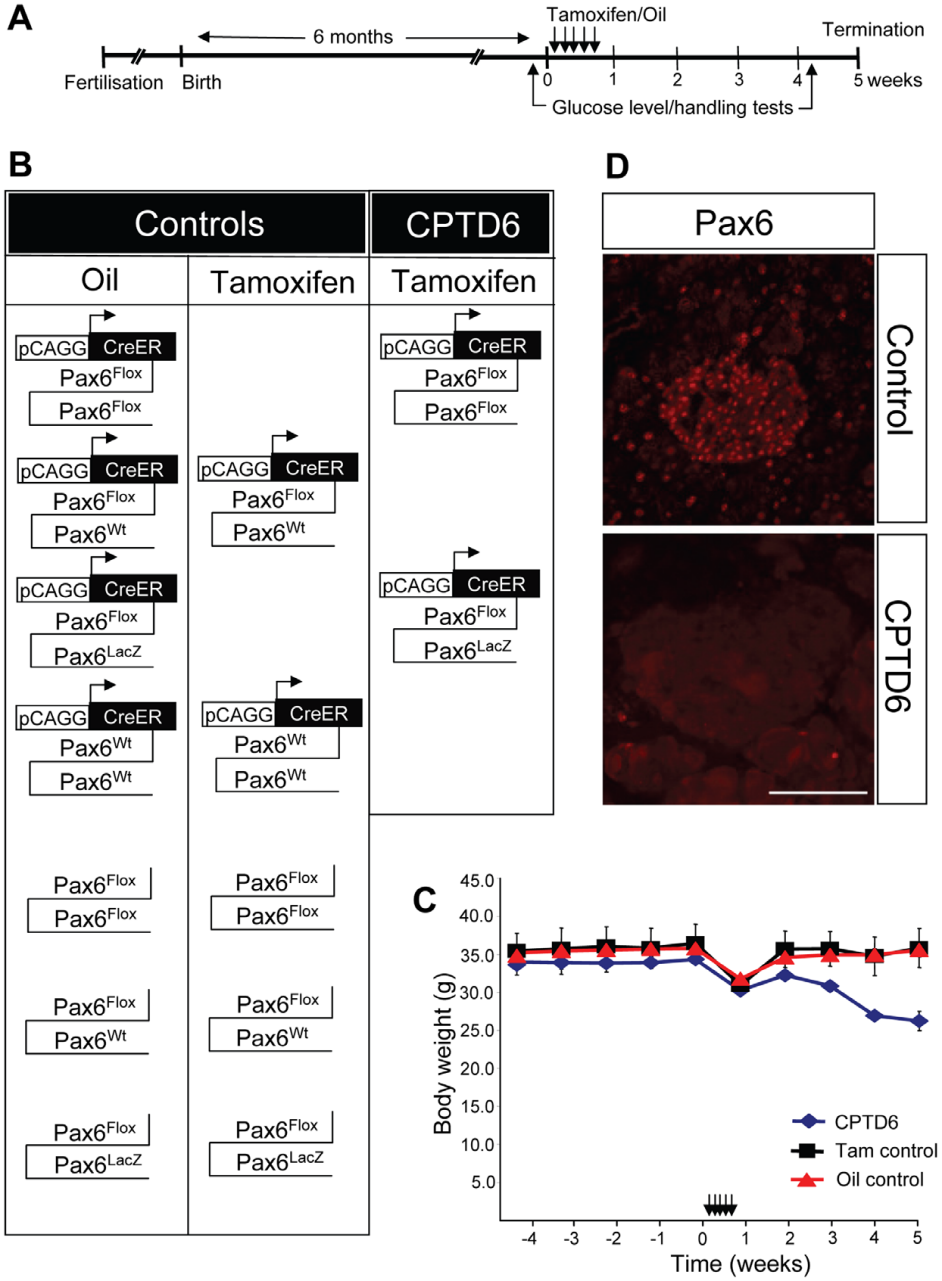
Experimental procedure used to investigate the role of Pax6 in the adult mouse pancreas. (**A**) Timeline of the experiment. Mice were bred to obtain the various experimental and control genotypes. After a pre-experimental period of 6 months (indicated by the period to the left of timepoint = 0 weeks) all mice received daily injections, with tamoxifen or oil only, on 5 consecutive days during week 1. Blood glucose level and glucose tolerance measurements were carried out in the week prior to the injections and in the final week (week 5). All mice were sacrificed at the end of week 5 and tissues were fixed for immunohistochemistry (**B**) Schematic representation of the combinations of genotypes and treatments (oil or tamoxifen injection) of all animals used in the study. Animals were sorted into control (oil only control or Tamoxifen control) or CPTD6 (CreER™-floxed Pax6-Tamoxifen-Diabetic) groups. (**C**) Graph showing male mouse body weights from 4 weeks pre-treatment until 5 weeks post-treatment, with a time-line of the procedure shown at the top. All animals exhibited healthy body weight prior to treatment. During the 5 day period of daily injections all animals lost some weight, but recovered in the week following the injections. While control animals continued to gain weight and were back to pre-treatment body weight at termination, CPTD6 animals began to lose weight again from the second week post-treatment onwards until termination of the experiment. Data are shown as mean ± SEM (Standard error of the mean). (**D**) Immunohistochemistry for Pax6 was used to assess the efficiency of the Cre-mediated deletion of *Pax6,* by staining for the presence or absence of Pax6. Control animals show nuclear Pax6 immuno-reactivity in the pancreatic islet cells. The CPTD6 islets show absence of Pax6 immuno staining in the large majority of cells, indicating successful Cre-mediated inactivation of the Pax6 gene. Scale bar, 50 µm.

The *Pax6^flox^* mice were kindly provided by Drs Ian Simpson and David Price. The *Pax6^flox^, Pax6^lacZ^* and *CAGGCreER™* transgenic lines have been published previously [6], [29], [30]. Mice were kept on CD1 or hybrid B6CBAF1 background. Figure 1 shows the combinations of alleles generated.

### Tamoxifen Injections

Intraperitoneal injections of corn oil only (control animals) or corn oil (glycerol trio-octonoate, Sigma C8267) containing dissolved Tamoxifen (Sigma T5648) (100 microgram/gram body weight) were administered to six month-old mice daily for 5 consecutive days. Prior to injection each animal was weighed and the injection volume of a 20 mg/ml tamoxifen stock solution or of corn oil alone adjusted accordingly.

### Glucose Tolerance Tests

Animals were fasted for five hours prior to intraperitoneal injection of 2 g glucose (Sigma) per kilogram of body weight. Blood was drawn from the tail vein at 0, 15, 30, 60, 90, and 120 minutes after injection and glucose values were monitored using an automatic glucometer (Accuchek, Roche).

### Urine Glucose Tests

Urine glucose levels were monitored weekly. Urine was collected from the animal and dropped onto a Diastix (Bayer) glucose stick. In accordance with the manufacturers’ instructions the result was recorded by photography after 30 seconds incubation. Darkening of the glucose sensitive area on the stick indicates an elevated urine glucose level that is compared to a standard series provided by the manufacturer.

### Immunofluorescence/immunohistochemistry

Tissues were fixed in 4% paraformaldehyde (PFA) for 3–6 hours at room temperature and then embedded in either paraffin or Tissue-Tek freezing solution. Sections were cut using a microtome or cryostat and 5–10µm sections were collected onto Superfrost slides. Microwave antigen retrieval with 10 mM Sodium Citrate (pH 6) was used to unmask the antigen prior to incubation in blocking solution of PBS/0.1% sheep serum. Primary antibodies were incubated overnight at 4°C and secondary antibodies for 60 minutes at room temperature. The following antibodies were used: Pax6 (Abcam), Insulin (Dako), Glucagon (Dako), Somatostatin (Dako), PC1/3 (Pcsk1; Enzo), PC2 (Pcsk2; Enzo), Isl1 (kind gift Helena Edlund), Pdx1 (Ipf1; kind gift Helena Edlund), Glut2 (Slc2a2; kind gift Helena Edlund), Ghrelin (Santa Cruz), Amylase (Sigma), Nkx2.2 (DSHB), Nkx6.1 (DSHB). Alexa594 conjugated secondary antibody (Invitrogen), FITC conjugated secondary antibody (Jackson ImmunoResearch).

### Quantification of Islet Cell Populations

Quantification of numbers of hormone producing cells relative to the total islet cell number was performed using the cell counter plug-in of Image J software (http://imagej.en.softonic.com/). Hormone expressing cells (insulin, glucagon, somatostatin and ghrelin) were visualised by their immunofluorescent marker and all cells stained above background were counted as positive. Total islet cell numbers were obtained by visualisation with DAPI staining. Cell numbers were counted on sections from at least 4 different control and CPTD animals each. Total cell numbers counted for presence of hormone expression were: Insulin (2209 control, 3398 CPTD6), Glucagon (2087 control, 2297 CPTD6), Somatostatin (4122 control, 4889 CPTD6), Ghrelin (2193 control, 3145 CPTD6). Raw cell counts were analysed in Excel and statistical analysis was done using a students t-test. Quantifications were performed on pancreatic sections from four different animals for each group (control and CPTD6).

## Results

### Generating Animals of Required Genotypes

The timeline of the experiment is schematically represented in figure 1A. To generate mice of the required genotypes floxed Pax6 animals (Pax6^flox/+^) were crossed with *CAGGCreER™* transgenic mice to generate Pax6^flox/+^//CAGGCreER™ mice. These were then crossed to heterozygous *Pax6^flox/+^* or *Pax6^LacZ/+^* mice to generate animals of the desired genotypes (Figure 1B). Animals were genotyped by PCR (details available on request). Two sets of controls were included: 1. “Oil control”, a set with the designated genotypes including animals with and without the *CAGGCreER™* transgene, injected with corn oil only. 2. “Tamoxifen control”, a second set containing heterozygous and wild-type mice, with and without the *CAGGCreER™* transgene, injected with Tamoxifen. The experimental group, designated “CPTD6”, consisted of Pax6^flox^/Pax6^flox^ or Pax6^flox^/Pax6^lacZ^ mice carrying the *CAGGCreER™* transgene and injected with Tamoxifen.

### Assessment of Initial Metabolic Status

**Figure 2:**
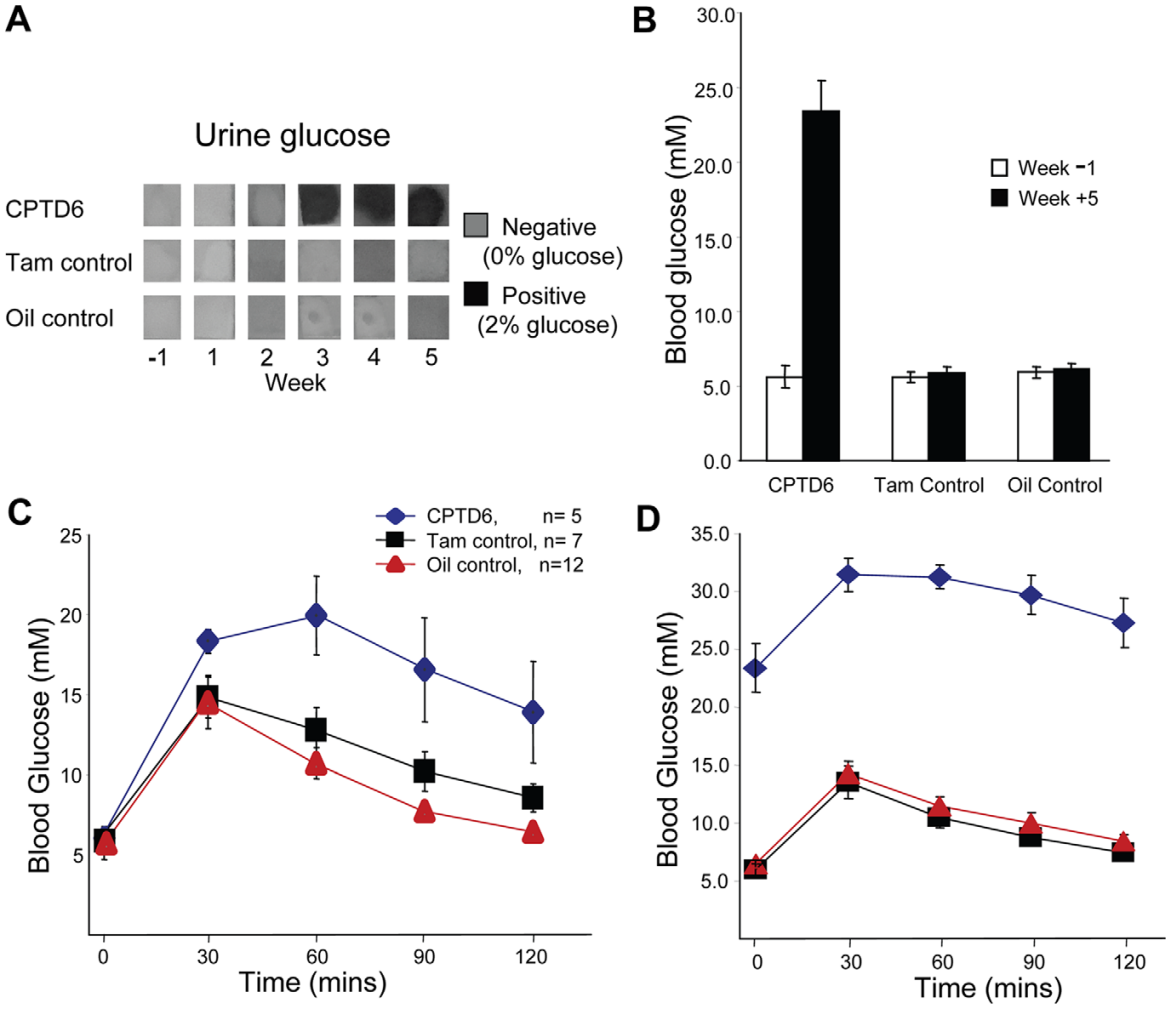
Assessment of glucose handling in control and CPTD6 mice. (**A**) Urine glucose levels were tested weekly using Diastix (Bayer) test sticks from week 0 (before first injection) to termination of the experiment (week 5). CPTD6 mice presented with glucose in their urine when tested 3 weeks post first injection. None of the control animals (tamoxifen- nor oil-injected controls) showed glucose in their urine at any time. (**B**) Fasting blood glucose levels were normal in all three experimental groups in the week prior to the tamoxifen/oil injections (white bars). When tested again in the final week of the experiment (start of week 5) CPTD6 mice presented with hyperglycaemia (black bars) (**C**). A glucose tolerance test performed in the week before injection revealed slight glucose intolerance in CPTD6 mice compared to the control animals. (**D**) Glucose intolerance of the CPTD6 mice was exacerbated post injection. Data are shown as mean ± SEM.

To obtain baseline data the animals were weighed on a weekly basis (Figure 1C) and their general health assessed from 4 weeks prior to injections. No significant difference in weight between the control and experimental groups (Figure 1C) was observed. In addition, one week prior to injection (Week -1), urine glucose measurement, fasting blood glucose and glucose tolerance tests were performed (Figure 2A, B, C). Glucose was not detected in urine samples from any animal in either the control or the experimental groups (Figure 2A). Fasting blood glucose levels were similar in both controls and CPTD6 mice (Figure 2B, white bars). However, the glucose tolerance test (Figure 2C) revealed slight intolerance in the CPTD6 group with the area under the curve approximately 1.5 fold higher than in control animals (n = 5, CPTD6, n = 12 Oil control, n = 7 Tamoxifen control). The animals in the CPTD6 group were a combination of *Pax6^flox^/Pax6^flox^//CAGGCreER™* and *Pax6^flox^*/*Pax6^lacZ^*//*CAGGCreER™*, but were predominantly the latter genotype (2+3), therefore carrying only one functional copy of *Pax6*, and this may account for this observation by itself, or could suggest a slight leakiness of the *CAGGCreER™* transgene to which *Pax6^flox^*/*Pax6^lacZ^* would be more susceptible.

Five daily intraperitoneal injections of corn oil or Tamoxifen (in corn oil) were administered, and animals were weighed daily at this stage. Animals in all three groups experienced a similar weight loss over this five day period (Figure 1C). Both control groups and the CPTD6 experimentals recovered and had gained several grams of body weight by 7 days after the last injection. The weight gain continued through the second and subsequent weeks for all control mice, however at 14 days after the last injection the CPTD6 mice were losing weight again. This weight loss in the CPTD6 mice continued until termination of the experiment 4 weeks after the last injection of Tamoxifen (Figure 1C).

### Effect of *Pax6* Inactivation on Glucose-handling

Non-fasting urine samples continued to be monitored from Week 1 (day of final injection), at weekly intervals. None of the control mice (Oil control and Tam control) had detectable levels of glucose in their urine at any stage. In contrast at week 3, just over two weeks after the last injection the CPTD6 mice presented with a positive urine glucose test, with concentrations greater than 2% (>11.1 mM; see Methods) which was maintained at week 4 and 5 (Figure 2A). Fasting blood glucose was measured at the beginning of week 5 followed by a glucose tolerance test. The CPTD6 mice presented with a mean elevated fasting blood glucose level of 23.4 mM compared with oil injected control mice with 5.9 mM and tamoxifen injected control mice with 6.4 mM (Fig. 2, n = 5, n = 12, n = 7, *P*<0.05) (Figure 2B, black bars). The elevated fasting blood glucose was accompanied by an impaired ability to clear glucose when challenged with glucose resulting in a 3 fold increased area under the glucose tolerance test curve of CPTD6 mice as compared to all control mice (Figure 2D, n = 5, n = 19). CPTD6 mice were also observed to drink and urinate more, indicating the CPTD6 animals presented all classical symptoms of diabetes: weight loss, polydypsia, polyuria, glycosuria and elevated fasting blood glucose levels.

### Immunohistochemical Analysis of Pancreas

All animals were sacrificed at the end of week 5 (i.e. 4 weeks after the final tamoxifen injections) and their pancreata fixed and analysed for expression of a number of pancreatic markers, including several pancreatic transcription factors, endocrine hormones and processing enzymes.

#### Pax6 expression was severely diminished

To assess the efficiency of the Cre mediated inactivation of the *Pax6* gene, the first marker to be tested was Pax6 itself (Fig. 1D), revealing a loss of detectable protein in most islet cells of the CPTD6 mice (n = 4). Some residual expression was observed in a small number of individual CPTD6 islet cells, while in controls (n = 4) clear nuclear staining was observed in all islet cells. These data show that the CreER™ protein was successfully activated by the tamoxifen treatment in the large majority of islet cells, resulting in excision of the floxed *Pax6* allele and loss of Pax6 production from the inactivated allele. DAPI staining of CPTD6 pancreata showed the distinct organisation of endocrine cells into islet-like structures was maintained, although many islets presented with a less organized, round/oval structure compared to the more elongated architecture seen in control islets.

#### Expression of endocrine hormones in CPTD6 islets

**Figure 3:**
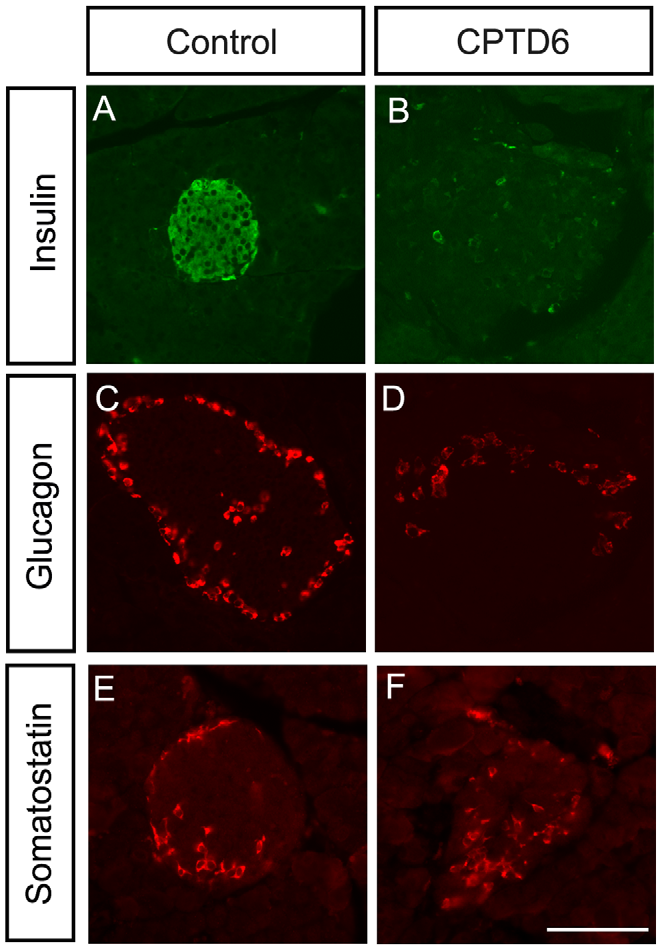
Expression analysis of pancreatic endocrine hormones. Images show immuno-stainings for insulin (**A**, **B**), glucagon (**C**, **D**), and somatostatin (**E, F**) in sections through the pancreas of control (**A**, **C**, **E**) and CPTD6 (**B**, **D**, **F**) mice sacrificed 5 weeks post-injections. Insulin expression is almost completely abolished in CPTD6 islets compared with control islets (**A, B**)**.** The number of cells staining positive for glucagon is also decreased in the CPTD6 islets compared to controls (**C, D**). Somatostatin expression is more moderately reduced in the CPTD6 islets with most CPTD6 islets showing fewer positive cells compared to the controls. (**E, F**). Images are representative of at least four animals from each group. Scale bar, 50 µm.

Immunohistochemical analysis revealed both insulin (Figure 3A, B) and glucagon (Figure 3C, D) expression were severely reduced in the CPTD6 animals. For both hormones the number of expressing cells was much reduced, and co-staining revealed altered islet architecture with α cells appearing in more internal positions in the CPTD6 islet (Figure 3C, D). Control islets show the normal islet architecture with β cells at the centre surrounded by a mantle of α cells. A moderate reduction in the number of somatostatin-expressing cells was also detected (Figure 3E, F).

#### Expression of key pancreatic transcription factors

**Figure 4:**
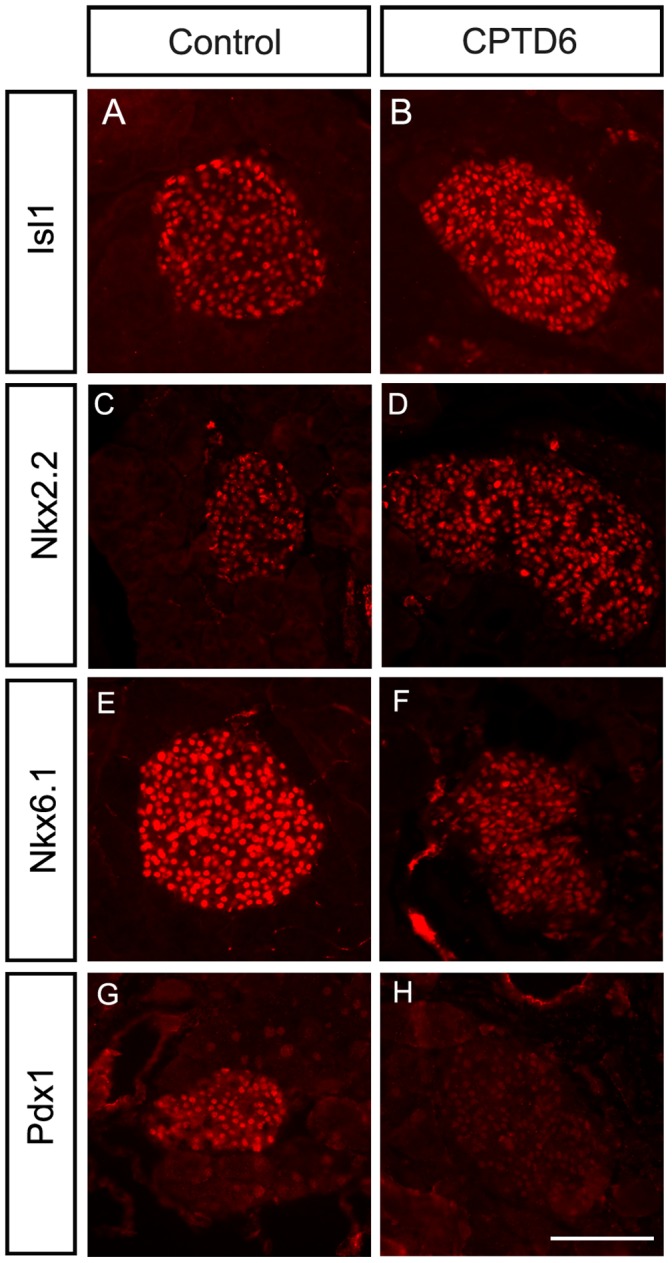
Expression analysis of key transcription factors. Immunohistochemistry was performed in control (**A**, **C**, **E**, **G**, **I, K**) and CPTD6 (**B**, **D**, **F**, **H**, **J, L**) mice. (**A**, **B**) Nuclear expression of the pancreatic islet marker Islet 1 (Isl1) is retained in CPTD6 mice, showing that the islet cells are present. (**C,D**) Expression of Nkx2.2 is also unaffected in CPTD6 mice. (**E, F**) In contrast expression of Nkx6.1 is reduced in Pax6 deficient cells. (**G, H**) The nuclear expression of the transcription factor Pdx1 is lost in CPTD6 mice. Images are representative of at least four animals from each group. Scale bars, 50 µm.

Expression of Islet1 (Isl1), an important regulator for pancreas development, showed no significant difference between control and CPTD6 mice and confirmed that islet cells were still present in the CPTD6 animals (Figure 4A, B). Similarly the expression of Nkx2.2 appeared unaffected in the CPTD6 islets (Figure 4C, D). In contrast, the adult β-cell marker Nkx6.1 (Figure 4E, F) was downregulated in the absence of Pax6. Interestingly, Pdx1, which is required for pancreas development and β-cell function, was also severely downregulated in the CPTD6 animals with very few islets showing prominent levels of Pdx1 expression (Figure 4G, H). As previous work has shown a requirement for Pdx1 expression in adult β-cells for maintenance of glucose sensing and the proinsulin-processing machinery [31], we next analysed the status of some key components [31].

#### Analysis of additional key islet cell markers

**Figure 5:**
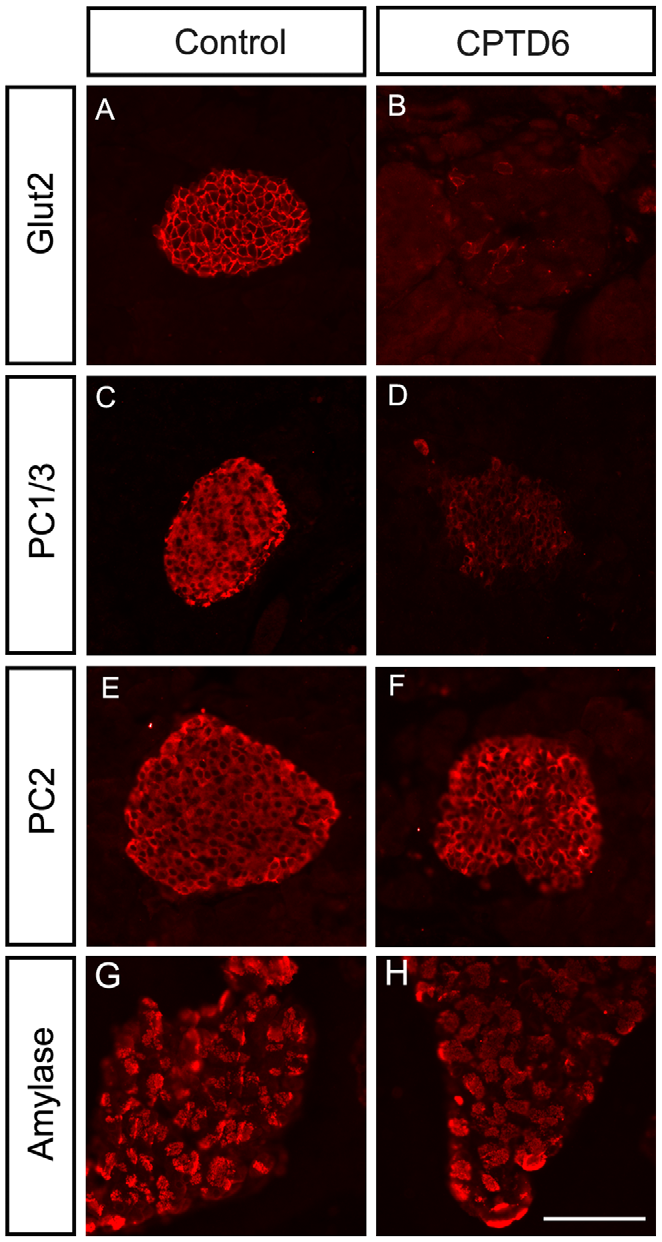
Glucose sensing, hormone processing and the exocrine compartment. (**A**, **B**) Expression of the glucose transporter Glut2 (Slc2a2), localised at the endocrine cell surface, is lost in CPTD6 mice. (**C, D**) Cytoplasmic expression of the prohormone processing factor PC1/3 (Pcsk1) is absent in CPTD6 mice. (**E**, **F**). Expression of the processing factor PC2 (Pcsk2) and (**G, H**) the exocrine enzyme amylase are not affected.

Glut2 (Slc2a2), a glucose transporter that is required post-prandial to sense glucose fluctuations, is expressed at the surface membrane of adult β-cells. Glut2 expression was almost completely abolished in the CPTD6 animals compared with controls (figure 5A, B). Insulin is processed from a larger precursor, proinsulin through a regulated secretory pathway and stored in secretory granules in readiness for secretion as active insulin. A number of proteins are required for proinsulin processing into its active disulphide bond linked A and B chains. Two of these, PC1/3 (Pcsk1) and PC2 (Pcsk2), act in concert with additional proteins to cleave proinsulin at specific dibasic residue junctions to produce equimolar amounts of active insulin and C-peptide. Immunohistochemical analysis of PC1/3 (figure 5C, D) revealed that expression of this enzyme is strongly reduced in the CPTD6 animals, although some cells within the islet maintain high levels of PC1/3 expression. In contrast, PC2 expression does not appear to be affected (Figure 5E, F).

#### Exocrine pancreas

Gross histology of the exocrine pancreas from CPTD6 mice suggested a slight difference from controls, showing a less organised and looser architecture with fewer acinar structures and a more prominent ductal network. Nevertheless, the CPTD6 exocrine pancreas showed normal positive staining for amylase expression (Figure 5G, H), presenting the same punctuate expression pattern in acinar structures as control pancreata (CPTD6, n = 4, controls, n = 4).

#### Strong upregulation of ghrelin in CPTD6 islets

**Figure 6:**
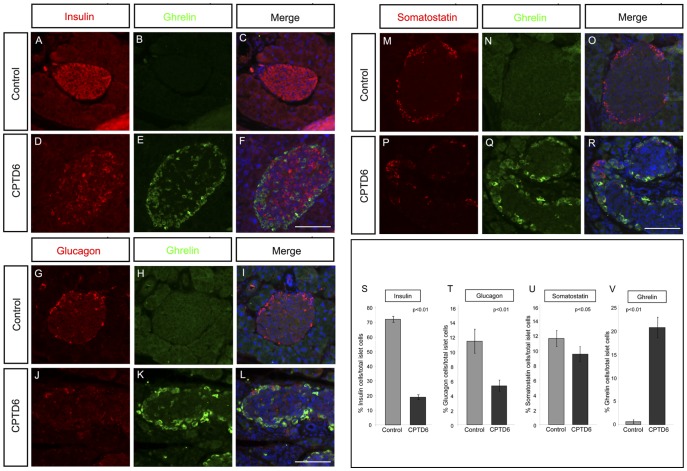
Upregulation of Ghrelin expression in CPTD6 islets. Immunohistochemistry for ghrelin (**B,** **E, H, K, N, Q**) was performed in combination with insulin (**A**, **D**), glucagon (**G, J**), and somatostatin (**M, P**). Merged images with DAPI nuclear counter-staining are shown (**C, F, I, L, O, R**) on pancreata from control (**A**, **B, C, G, H, I, M, N, O**) and CPTD6 (**D, E, F, J, K, L, P, Q, R**) mice. Levels of insulin and glucagon expression are markedly reduced or lost in the majority of islet cells of CPTD6 mice in comparison with the controls, while the number of somatostatin positive cells appears more moderately reduced. Very few, if any, ghrelin positive cells can be found in the islets of control adult mice (**B, C**, **H, I, N, O**)**.** In contrast, strong staining is observed in many cells of the CPTD6 pancreata, located predominantly around the periphery of the islets. Images are representative of at least four animals from each group. Scale bars, 50 µm. (S, T, U, V). Quantification of the relative numbers of hormone expressing cells in the islets of Langerhans of control and CPTD6 animals shown as the percentage of immunofluorescence positive cells per total number of DAPI positive cells. (S) Insulin producing cells (control 72% >19% CPTD6), (T) Glucagon producing cells (control 11.4% >5.3% CPTD6) (U) Somatostatin producing cells (control 11.6% >9.5% CPTD6), and (V) Ghrelin producing cells (control 0.6% >20.6% CPTD6).

In contrast to the loss of classical hormone production in α, β and δ cells (Figure 6A, D, G, J, M, P), a large increase in ghrelin positive cells was observed in CPTD6 islets. Ghrelin positive cells are very rare or absent in a normal adult pancreas, as illustrated by the controls studied here (Figure 6B, H, N), but the CPTD6 islets exhibit large numbers of ghrelin positive cells, located predominantly in the periphery of the islets (Figure 6E, K, Q). Co-expression studies for ghrelin with insulin, glucagon or somatostatin (Figure 6C, F, I, L, O, R) reveal that the ghrelin positive cells do not express any of these three hormones, and should therefore be classified as ε cells. To obtain a quantitative measure of the differences in the numbers of hormone producing cells in the pancreatic islets of CPTD6 and control animals, the percentage of hormone expressing cells per islet was determined. Hormone expressing cells were identified by immunofluorescence and counted relative to the total number of DAPI positive cells in the islet. The average percentage of insulin positive cells per islet was decreased from 72% in the controls to 19% in CPTD6 animals, while the percentage of glucagon expressing cells decreased from 11.4% to 5.3%. A more modest change was seen in somatostatin positive cell numbers (11.6% in controls versus 9.5% in CPTD6). In contrast the percentage of ghrelin expressing cells showed a large increase from 0.6% in controls to 20.6% in CPTD6 pancreatic islets.

## Discussion

The developmental regulator Pax6 has a well-established role in the embryonic development of the endocrine pancreas and the correct differentiation of the different cell types in the islets of Langerhans. The data presented here show that there is a continued requirement for Pax6 in the adult to maintain a fully functioning endocrine pancreas. Pax6 is essential for development of the eye and brain, and homozygous loss of function results in neonatal lethality, precluding the analysis of the potential importance of Pax6 gene function in the tissues in which it remains expressed in adulthood. To bypass this problem we have studied the effect of loss of Pax6 in adult mice in a conditional Pax6 deletion model crossed with a tamoxifen inducible Cre deletor strain. In this way we were able to remove most functional Pax6 in mice that had lived a healthy life for the 6 months leading up to the experiments. Remarkably the deletion of Pax6 led to a rapid development of diabetic symptoms and deterioration of health in the homozygous Pax6 conditional mice that were Cre positive and treated with tamoxifen (named CPTD6 mice). Diabetic symptoms were not found in any control animals, except in one of the heterozygous Pax6 conditional, Cre-positive tamoxifen treated females, supporting the possibility of increased diabetes susceptibility in PAX6 heterozygous aniridia patients [20], [27], [32]. In the four weeks following Pax6 inactivation no obvious neurological, olfactory or eye abnormalities were observed, but more detailed histological analysis of some of these tissues is in progress.

We show that production of the endocrine hormones insulin, glucagon and somatostatin is strongly reduced in response to the adult stage inactivation of Pax6. The direct regulation of these hormones by Pax6 in the developing and neonatal pancreas is well-established [7], [12], and specific Pax6 binding sites have been described in the regulatory regions of these genes [7], [12], [33], [34]. Pax6 expression in adult islets of Langerhans has also been documented [19], [21], and is confirmed by our data, which demonstrates that the continued presence of Pax6 is essential for endocrine hormone production. There is increasingly convincing evidence that glucose handling and insulin production are also disturbed in adult rodents and humans who carry heterozygous PAX6 mutations [19], [20], [21], [28]. This suggests that not only is PAX6 essential for pancreatic endocrine maintenance, but that correct dosage of the protein is required for this role, as it is for some other transcription factors associated with diabetes, such as HNF1A and HNF4A, in which heterozygous mutations have been found in a significant portion of MODY cases [35].

The islet markers Isl1 and Nkx2.2 retain a normal expression pattern in the Pax6 deleted pancreas by immunohistochemistry, indicating there is no gross morphological change in the islets following Pax6 removal. Interestingly, we observe loss of Pdx1 and Nkx6.1 expression in the Pax6 deleted pancreata, indicating Pax6 acts upstream of these genes in adult pancreas. In contrast, during embryonic development Pdx1 acts upstream of Pax6 [9], [36], and no Pax6 expression is detected in the pancreas of Pdx1 knock-out mice [9]. Developmental regulators are well known to cross-regulate in reciprocal feedback loops in many systems and, while at this stage it is unclear whether Pdx1 is a direct Pax6 target, Pax6 and Pdx1 may be tightly connected through reciprocal wiring into the gene regulatory network for pancreas development and maintenance [37].

Previous work has shown that loss of Pdx1 expression in adult beta-cells disrupts key proteins involved in glucose sensing, including Glut2 (Slc2a2) and the proinsulin processing machinery, PC1/3 and PC2 [31]. Loss of Pax6 expression in the adult pancreas, as shown here, also affects these functions. Mice with a Glut2 null mutation develop diabetes [38], demonstrating the requirement for this sugar transporter. It is unclear whether the near total loss of Glut2 expression in our experimental animals is due directly to loss of Pax6 activity or as an indirect consequence of losing Pdx1 expression. Gosmain *et al* identified putative consensus Pax6 binding sites upstream of the Glut2 start site by *in silico* prediction, suggesting Pax6 could regulate Glut2 directly, but no binding was found by EMSA [34], and Pax6 binding site predictions by Hidden Markov Modelling did not reveal Glut2 as a target gene [39]. Chromatin immunoprecipitation studies with Pax6 and Pdx1 antibodies in pancreatic islet cells will be required to resolve this issue.

Another feature of type 2 diabetes is hyperpronisulinaemia [40] which often reflects a defect in the proinsulin processing pathway. Prohormone convertases PC1/3 and PC2 together with Carboxypeptidase E catalyse this cleavage sequence [41]. Expression levels of PC1/3 were severely reduced in Pax6 null animals, but PC2 was less affected, a situation reminiscent to that observed in the FRID1 mice and conditional Pdx1 null mice [31], and this suggests that Pax6 may directly regulate components of the insulin processing machinery, or could do so indirectly through regulation of Pdx1 and/or components of the FGF signaling family. Our *in vivo* observations are in agreement with data obtained in a recent study using Pax6 RNAi in a rat β-cell model [34], although we observed little, or no difference in PC2 expression, while a moderate reduction was reported in the rat β-cell model. Aniridia patients with *PAX6* mutations have been reported to have elevated proinsulin levels, even when not frankly diabetic; and PC1/3 levels are reduced in heterozygous *Pax6* mutant mice, with some evidence for direct binding of Pax6 to upstream elements of *PC1/3* [20], [42]. In contrast, it is suggested that PC2 may only be an indirect target of Pax6 [12], [43], but it is interesting to note that PC2 expression was significantly reduced in one Pax6 mutant mouse line [44].

In contrast to the near complete loss in insulin expression, severe reduction in glucagon- and moderate reduction in somatostatin positive cells, we observed a striking increase in ghrelin-expressing cells. The appearance of ghrelin producing cells in the Pax6-null islets of the CPTD6 mice suggests that Pax6 may be implicated in the control of ghrelin expression in the adult pancreas. Reduction in insulin and glucagon expressing cells has also been observed when Pax6 is inactivated during development, both in Pax6 mutants and in a pancreas specific knock-out of the gene [44], [45]. Dames et al. also observed an increase in ghrelin positive cells and a quantitatively similar reduction in glucagon positive cells and suggested a developmental lineage switch of alpha to epsilon cells in the absence of Pax6. It is difficult to say whether the ghrelin-positive cells, observed when Pax6 is turned off in adulthood, are derived by transdifferentiation from alpha cells. The majority of the newly ghrelin-expressing cells are positioned at the periphery of the islet, like alpha cells, but further analysis is required to determine the origin of these cells. Ghrelin is predominantly expressed in the stomach in adulthood and is involved in regulating food intake, hepatic glucose production and preventing glucose disposal in muscle and adipose tissue [46]. The predicted increase in ghrelin production, coupled with the marked reduction in insulin expression, may explain the rapid onset of diabetes in our animals [47]. Animals in which either the ghrelin gene or the ghrelin receptor has been silenced have improved glucose tolerance [48], [49], [50], indicating a distinct role for ghrelin in glucose homeostasis.

Our studies add an adult requirement for Pax6 to its well-known developmental role [4], [5]. The scale of its many pleiotropic functions is reflected in the complex cis-regulatory architecture of the Pax6 genomic locus [51]–[55]. While the essential role of Pax6 for embryonic development and post-natal viability obscures its role in adult maintenance of target organs, and its potential involvement in adult-onset diseases such as diabetes, this role would be unmasked by mutations or variations in the cis-elements that drive Pax6 expression in these adult tissues. Currently the cis-elements required for expression in the adult pancreas are unknown, but following our demonstration of the essential role for Pax6 in pancreas homeostasis, their identification has gained greater importance. Two elements located upstream of the Pax6 P0 promoter are known to drive expression in the pancreas during embryonic development [51], [53], [54], but it is unknown if these remain active during adult life, and adult expression may well depend on further cis-elements in the locus. Sequence variation in these elements could constitute a risk factor for diabetes [42].

In conclusion, we show that loss of Pax6 in the adult islet affects the expression of multiple target genes involved in the maintenance of pancreatic endocrine function and glucose handling, and results in the rapid appearance of diabetic symptoms in previously healthy mice.
